# Tadalafil Nanoemulsion Mists for Treatment of Pediatric Pulmonary Hypertension via Nebulization

**DOI:** 10.3390/pharmaceutics14122717

**Published:** 2022-12-05

**Authors:** Bassant Elbardisy, Nabila Boraie, Sally Galal

**Affiliations:** 1 Department of Pharmaceutics, Faculty of Pharmacy, Alexandria University, Alexandria 21521, Egypt; 2 Institute for Technical Chemistry, Braunschweig University of Technology, Hagenring 30, 38106 Braunschweig, Germany

**Keywords:** Tadalafil, pulmonary hypertension, nebulization, nanoemulsion, phosphodiesterase 5 inhibitor, oro-tracheal, aqueous dilution, pulmonary drug delivery, pediatric

## Abstract

Oral tadalafil (TD) proved promising in treating pediatric pulmonary arterial hypertension (PAH). However, to ensure higher efficacy and reduce the systemic side effects, targeted delivery to the lungs through nebulization was proposed as an alternative approach. This poorly soluble drug was previously dissolved in nanoemulsions (NEs). However, the formulations could not resist aqueous dilution, which precluded its dilution with saline for nebulization. Thus, the current study aimed to modify the previous systems into dilutable TD-NEs and assess their suitability for a pulmonary application. In this regard, screening of various excipients was conducted to optimize the former systems; different formulations were selected and characterized in terms of physicochemical properties, nebulization performance, stability following sterilization, and biocompatibility. Results showed that the optimal system comprised of Capmul-MCM-EP:Labrafac-lipophile (1:1) (*w*/*w*) as oil, Labrasol:Poloxamer-407 (2:1) (*w*/*w*) as surfactant mixture (S_mix_) and water. The optimum formulation P2_TD_ resisted aqueous dilution, exhibited reasonable drug loading (2.45 mg/mL) and globule size (25.04 nm), acceptable pH and viscosity for pulmonary administration, and could be aerosolized using a jet nebulizer. Moreover, P2_TD_ demonstrated stability following sterilization and a favorable safety profile confirmed by both in-vitro and in-vivo toxicity studies. These favorable findings make P2_TD_ promising for the treatment of pediatric PAH.

## 1. Introduction

Pulmonary arterial hypertension (PAH) is a progressive cardiopulmonary disease that interferes with the ability of patients to exercise or perform routine duties [1]. According to the 6th World Symposium on Pulmonary Hypertension in France in 2018, PAH occurs when the mean pulmonary artery pressure (mPAP) exceeds 20 mmHg at rest [2]. Pathogenesis of PAH includes vascular remodeling giving rise to narrow pulmonary arteries, increasing pulmonary vascular resistance (PVR), leading to right ventricular failure and death [2]. PAH is a multi-factorial disease due to its complex etiology [1]. Symptoms of the disease include nonproductive cough, dyspnea, syncope, fatigue, and peripheral edema [1]. Pediatric PAH is increasingly recognized to have different etiologies, clinical presentations, and outcomes than adult PAH [3]. In 2010–2013, the incidence rate of pediatric PAH in the United States was estimated to be 4.8 to 8.1 per million children a year, and the annual prevalence ranged from 25.7 to 32.6 per million children [3]. The management of Pediatric PAH is still challenging despite the recent advances since the pediatric population possesses variable growth, development, and metabolism characteristics, which complicates the extrapolation of conclusions from the adult data to pediatrics [2].

A number of physiological pathways are involved in the pathogenesis of PAH, including the endothelin, nitric oxide (NO), and prostacyclin pathways [3]. Accordingly, various drug classes based on targeted pulmonary vasodilator therapy demonstrated beneficial therapeutic efficacy via hemodynamic and functional improvement, including (i) endothelin receptor antagonists, (ii) phosphodiesterase 5 inhibitors (PDE5-Is), and (iii) and prostacyclin analogs [2]. PDE5-Is exert their vasodilating effect in the pulmonary vasculature through inhibition of phosphodiesterase 5 (PDE5), the enzyme that breaks down the secondary messenger cyclic guanosine monophosphate (cGMP), which regulates smooth muscle contraction, resulting eventually in vasodilatation [3]. Among the available PDE5-Is, both sildenafil and tadalafil (TD) were approved by the Food and Drug Administration (FDA) for the treatment of PAH in adults, as these medications were shown to enhance the exercise capacity and delay the clinical worsening of the case [2].

On the other hand, neither of the two drugs was approved by the FDA for treating PAH in children [2]. Despite the extensive use of sildenafil as an off-label cure for pediatric PAH since 2005 [4], the FDA released a strong warning in August 2012 against its chronic use for children aged 1–17 years [2]. This warning was announced after a long-term clinical study of oral sildenafil (STARTS-2), which proved that children administered a high dose of sildenafil are at higher risk of death, while those receiving low doses do not demonstrate obvious improvement in the exercise ability [2]. Therefore, much attention was directed to TD for treating PAH in children. TD offers many advantages for treating PAH in children, where it is a long-acting PDE5-I administered once daily [2]. Furthermore, reported data showed that it could be well tolerated by children, as it offers a favorable adverse effect profile and provides better clinical improvement than sildenafil [5].

Recent reports demonstrated that many therapies developed for treating PAH were delivered through the pulmonary route [6]. Concerning the delivery of PDE5-Is, the oral route is associated with a number of adverse effects, such as headache, flushing, rare systemic hypotension, erection, and transient color vision disturbance [2]. In the current study, the pulmonary route has been proposed for delivery of TD via nebulization for treatment of pediatric PAH, as direct delivery of medications to the lungs via inhalation is believed to offer many advantages, such as the local delivery provides direct access to the airways; thus high drug concentrations can be attained in the target organ, ensuring high efficacy [6]. In addition, rapid drug absorption is secured via this route of administration due to the high vascularization, huge absorptive lung surface area, and excellent permeability, resulting in a fast onset of action [6]. The targeted drug delivery reduces the systemic side effects and offers the potential to reduce both the medication dose and the frequency of administration, thus improving patient compliance [6]. Moreover, pulmonary drug delivery avoids first-pass metabolism due to the relatively low enzymatic activity of the lungs [6].

Many efforts have been made to overcome the limitations accompanying the conventional treatment using anti-PAH drugs and improve their efficacy through employing novel drug delivery systems, including liposomes, polymeric nanoparticles, solid lipid nanoparticles (SLNs), nanostructured lipid carriers (NLCs), micelles and nano-erythrosomes [6,7]. However, nanoemulsions (NEs) were chosen in the current study as they proved efficacy in the pulmonary delivery of poorly soluble drugs [8,9,10,11,12,13,14,15,16]. Since TD is a poorly soluble class II drug [17], whose bioavailability is greatly affected by its rate and extent of dissolution, NEs were proposed as the delivery system of choice due to their high drug solubilizing capacity [10] and different forms of emulsions manifested superior solubilization of TD [18,19,20,21,22]. NEs are heterogeneous colloidal systems of oil and aqueous medium stabilized by surfactant (SAA) molecules [23]. These systems are kinetically stable during long-term storage without apparent flocculation or coalescence owing to their nanometer-sized droplets [23]. The reduced droplet size (<100 nm) is also advantageous for improving the drug bioavailability via facilitating mucosal drug penetration [10]. In addition, concerning pulmonary drug delivery, it was reported that the inhalation and aerosolization performances of NEs are superior to those of commercial suspensions and other related formulations, such as nanoparticles, liposomes, and micelles [10].

Despite the aforementioned merits of NEs, many systems are destroyed upon dilution with the aqueous phase, followed by drug precipitation and uncontrolled absorption [24]. Aqueous dilution with saline is required before nebulization to adjust the tonicity of the formulations and prevent aerosol-induced cough [25]. Our research group successfully developed intranasal TD-NEs for the treatment of erectile dysfunction based on the following ingredients (System A): Capmul-MCM-EP, Labrasol, Transcutol HP, and water [17], but unfortunately, these NEs turn turbid upon aqueous dilution, followed by precipitation of the dissolved drug.

On this basis, the current work endeavors for the first time—to the best of our knowledge—to develop TD-NEs that can resist aqueous dilution to be used for the treatment of pediatric PAH via nebulization using a jet nebulizer. Nanoparticles, microspheres, and nanocomposites of TD have been previously developed as inhalable dry powders for treating PAH [26,27,28]. However, it has been reported that dry powder formulations encounter problems during powder processing and re-dispersion (adhesion and strong agglomeration), which makes them unsuitable for efficient inhalation [10]. On the other hand, the administration of liquid formulations via nebulization is much more convenient, where the generated aerosol enables deep deposition in the lower respiratory tract [29].

In the context of the study objectives, several tests were carried out to modulate system A into a more stable system via screening various excipients. Since high excipient concentration in the formulation can induce dose-related irritancy or toxicity problems during pulmonary administration [11], the screening method aimed to select the NE excipients at their minimal optimal concentration. Then, different formulations of variable composition were fabricated and optimized via assessment of their physicochemical properties in drug solubilization, pH, viscosity, globule size, zeta potential, morphology, and stability. In addition, the suitability of the engineered formulations for pulmonary application was evaluated by studying nebulization, sterilization, and in-vitro and in-vivo toxicity studies.

## 2. Materials and Methods

### 2.1. Materials

Tadalafil (TD) was purchased from Radiant Pharma (Mumbai, India). Labrasol^®^ (Caprylocaproyl macrogol-8 glycerides EP, hydrophilic–lipophilic balance (HLB) = 14), Transcutol^®^ HP (Highly purified Diethylene glycol monoethyl ether EP/NF) and Labrafac^TM^-lipophile WL1349 (Medium chain fatty acid triglyceride JPE) were kind gifts from Gattefossé (Lyon, France). Capmul^®^ MCM EP (Glycerol monocaprylocaprate, a mixture of medium chain length monoglyceride (60%) and diglyceride (35%), consisting of 83% *w*/*w* caprylic acid (C_8_) and 17% *w*/*w* capric acid (C_10_)) was donated as a gift from Abitec Corporation (Janesville, WI, USA). Cremophor^®^ EL (Polyoxyl 35 castor oil USP, HLB = 12–14), Cremophor^®^ RH40 (Polyoxyl 40 hydrogenated castor oil USP/NF, HLB = 14–16), and Poloxamer-407 (Lutrol^®^ F 127, HLB = 18–23) was obtained from BASF (Ludwigshafen, Germany). Tween-80 (HLB = 15) and propylene glycol were provided by El-Nasr Pharmaceutical Chemicals (Cairo, Egypt). Polyethylene glycol 400 (PEG 400) was purchased from Sisco Research Laboratories Pvt. Ltd. (Mumbai, India). Human lung adenocarcinoma epithelial cell line (A549) was obtained from the American Type Culture Collection (ATCC) (Saint Cloud, MN, USA) through the Tissue Culture Unit, Egyptian Organization for Biological Products and Vaccines, Vacsera (Cairo, Egypt). Dulbecco’s Modified Eagle’s Medium (DMEM) was purchased from Lonza (Biowhittaker, Belgium) supplemented with 10% fetal bovine serum (FBS) (Lonza, Biowhittaker, Belgium), 1% (*v*/*v*) L-glutamine (Lonza, Biowhittaker, Belgium) and 1 g/L glucose (Lonza, Biowhittaker, Belgium). 3-(4,5-Dimethylthiazol-2-yl)-2,5-diphenyl-tetrazolium bromide (MTT) was purchased from Serva (Heidelberg, Germany). Ketamine^®^ (Ketamine hydrochloride 50 mg/mL) was purchased from Sigmatec Pharmaceutical Industries (Giza, Egypt). Xyla-ject^®^ (Xylazine hydrochloride 20 mg/mL) was purchased from Adwia Pharmaceuticals (Cairo, Egypt). Lactate dehydrogenase (LDH) and protein cytotoxicity detection kits were obtained from Roche Diagnostics (Indianapolis, IN, USA). All other chemicals and reagents were of analytical grade.

### 2.2. Screening of Surfactants (SAAs) and Co-Surfactants (Co-SAAs): Evaluation of Dispersion Properties

In order to produce a stable NE which can resist dilution with water, the nanoemulsifying properties of different SAAs and Co-SAAs were evaluated by visual assessment. The oily phase was mixed with the SAAs or SAA mixtures (S_mixs_) in a 1:3 (*w*/*w*) ratio, heated in a water bath at 40–50 °C and mixed with the aid of a vortex mixer (VM-300, Gemmy Industrial Corp., Taipei, Taiwan) to form a homogenous mixture. The oil-SAA mixture, 250 mg, was dispersed into 5 mL of distilled water with gentle mixing. Nanoemulsification was evaluated by visual observation of the final mixture (clear/turbid) after being left overnight at room temperature (RT) for equilibrium. Co-SAAs were screened by mixing the selected S_mix_ with the Co-SAA in a 2:1 (*w*/*w*) ratio, followed by the addition of the oily phase to this mixture in 1:3 (*w*/*w*) ratio, heating, and vortex to obtain a homogenous mixture. They were assessed in the same manner as explained before.

### 2.3. Construction of Pseudo-Ternary Phase Diagrams

Pseudo-ternary phase diagrams of two systems (B and C) were developed to identify the NE existence region via employing the modified titration method. The following combination of ingredients was used in the preparation of these systems:

- System B: Capmul-MCM-EP:Labrafac-lipophile WL 1349 (1:1) (*w*/*w*) as oily phase, Labrasol:Cremophor EL (1:1) (*w*/*w*) as S_mix_ and water as the aqueous phase.
- System C: Capmul-MCM-EP:Labrafac-lipophile WL 1349 (1:1) (*w*/*w*) as oily phase, Labrasol:Poloxamer-407 (2:1) (*w*/*w*) as S_mix_ and water as the aqueous phase. The (2:1) (*w*/*w*) S_mix_ of Labrasol with Poloxamer-407 was prepared by heating the two ingredients until a homogenous mixture was obtained. A S_mix_ of equal weights of Labrasol and Poloxamer-407 was not employed as a S_mix_ due to the high viscosity of the mixture and the difficulty in withdrawing an accurate volume.

In transparent glass vials, nine dissimilar combinations of oily phase and water (1:9, 2:8, 3:7, 4:6, 5:5, 6:4, 7:3, 8:2, 9:1) were prepared on a weight basis, and they were slowly titrated with the selected S_mix_ under continuous stirring at RT till reaching clarity of the system. Afterward, the systems were allowed to equilibrate overnight at RT and were checked visually for clarity. In addition, their percentage transmittance (%T) was measured at 630 nm using ELx800^TM^ Absorbance Microplate Reader (BioTek Instruments Inc., Winooski, VT, USA) against distilled water as a blank. The amount of S_mix_ added till an opaque/transparent transition occurred was used to determine the phase domains. Phase diagrams were mapped using Sigma Plot^®^ software (version 13, Systat Software Inc., San Jose, CA, USA).

### 2.4. Preparation of TD-Loaded NE Formulations

#### 2.4.1. Preparation of Placebo NEs

NE formulations of various compositions were selected from the phase diagrams for further studies. Placebo NEs were formed spontaneously upon mixing the calculated weights of the oily phase, aqueous phase, and SAAs. Formulations of system B were formed at RT, whereas those of system C were prepared by dissolving the calculated weight of Poloxamer-407 in the calculated weight of water in an ice bath, followed by the addition of Labrasol under continuous stirring, then the oil mixture was added at the end. The stability of NEs towards aqueous dilution was evaluated by visual inspection of the clarity of the formulations following 50-fold dilution with distilled water after 2-h storage at RT.

#### 2.4.2. Rationale for Dose Calculation

The dose of TD to be administered via nebulization for the treatment of pediatric PAH was calculated following an example reported by Sawatdee et al. in which the blood concentration was multiplied by the extravascular lung fluid volume [30]. The pharmacokinetics of TD following a single oral dose of 40 mg in healthy adult male Japanese and Caucasian volunteers displayed a C_max_ of 446 ng/mL and 562 ng/mL, respectively [31]. The mean value of C_max_ for both races was 504 ng/mL. The reported lung fluid volume was 3–10 mL [30]. By considering both values, the TD dose varied between 1.5 µg and 5.04 µg. The percentage of nebulized dose delivered to the lung was reported to be around 10%, up to 30% in some reports [32]. In this estimation, the percentage of nebulized doses reaching the lungs was 10%. So, the adult dose of TD required for treating PAH through nebulization ranged between 15 µg and 50.4 µg. Half the adult dose was considered in the current investigation for the treatment of pediatric PAH. Therefore, the calculated dose of TD for the treatment of pediatric PAH via nebulization ranged between 7.5 µg to 25.2 µg.

#### 2.4.3. Determination of Drug-Loading Capacity of NEs

An excess amount of TD was dissolved in the oil/SAA phase of NEs by sonication (Elmasonic S 40 (H), Elma-Hans Schmidbauer GmbH & Co. KG, Singen, Germany) at ambient temperature (30 ± 2 °C) until no more drug dissolves, then the calculated weight of water was added to each mixture. Afterward, the prepared NEs were left for 24 h for equilibrium at ambient temperature, filtered through 0.45 µm cellulose acetate syringe filter (Minisart^®^, Sartorius, Goettingen, Germany), and the filtrates were properly diluted with methanol. The amount of drug dissolved was analyzed spectrophotometrically (UV-160A, Shimadzu, Kyoto, Japan) at λ_max_ 285 nm against a blank. TD-loaded formulations were prepared by dissolving TD in pre-formed placebo NEs to produce an NE of a final drug concentration of 2 mg/mL.

#### 2.4.4. Dispersion Stability of TD-Loaded NEs

The equilibrium solubility of TD was determined after 50-fold dilution of the drug-loaded NEs with distilled water at RT. An aliquot was taken immediately after dilution, followed by compensation with an equal volume, then another sample was withdrawn after 2 h to test for drug precipitation. TD concentration was measured spectrophotometrically after filtration and dilution with methanol. The amount of drug remaining after 2h dilution was calculated as a percentage relative to the original concentration determined after immediate dilution.

### 2.5. Physicochemical Characterization of Selected NE Formulations

The isotropic nature of NEs was confirmed using cross-polarized light microscopy (Olympus CX31PF, Olympus Corporation, Tokyo, Japan). Transparency of NEs was verified by measuring the refractive index using Abbe refractometer (Abbe 5 refractometer, Bellingham + Stanley Ltd., Kent, UK) and %T at 630 nm using ELx800^TM^ Absorbance Microplate Reader (BioTek Instruments Inc., Winooski, VT, USA). The pH of the formulations was measured at 25 ± 2 °C using a calibrated pH meter (Mettler-Toledo S220 SevenCompact™ pH/Ion meters, Mettler Toledo Co, Greifensee, Switzerland) after 50-fold dilution with saline. The viscosity of 50-fold saline-diluted NEs was measured at 25 ± 2 °C using a Brookfield viscometer (Brookfield Engineering Laboratories Inc., Stoughton, MA, USA) equipped with spindle 2 at 100 rpm. Globule size and polydispersity index (PdI) of the formulations were determined using Malvern zetasizer Nano ZS (Malvern Instruments, Malvern, UK) after 50-fold dilution with either water or saline. Zeta potential was measured using Malvern zetasizer Nano ZS (Malvern Instruments, Malvern, UK) after 50-fold dilution with saline. The morphology of TD-loaded NEs was studied using transmission electron microscopy (TEM) (model JEM-100CX, JEOL, Tokyo, Japan) after 50-fold dilution with saline. The thermodynamic stability of TD-loaded NEs was evaluated via accelerated stability studies in terms of physical stability (phase separation, creaming, cracking, and drug precipitation) and globule size after subjecting the NEs to centrifugation (4000 rpm for 15 min) and three freeze-thaw cycles (alternating cycles of storage at −4 °C and +25 °C for 24 h at each temperature).

### 2.6. Nebulization Performance of TD-Loaded NEs

An aliquot (4 mL) of 50-fold saline-diluted TD-loaded NEs was placed in the nebulizer cup of a Microlux^®^ jet nebulizer (AS109N, Medel, Parma, Italy) and nebulized for 20 min. The stability of NEs towards nebulization was assessed in terms of globule size and morphology using TEM. In addition, the aerosol output and aerosol output rate were determined on a gravimetric basis using the following equations [10]:

$$\mathrm{Aerosol}\;\mathrm{output}\;\left(\%\right)=\frac{\mathrm{Weight}\;\mathrm{of}\;\mathrm{nebulized}\;\mathrm{liquid}\;\left(g\right)}{\mathrm{Weight}\;\mathrm{of}\;\mathrm{liquid}\;\mathrm{before}\;\mathrm{nebulization}\;\left(g\right)}\times 100$$

$$\mathrm{Aerosol}\;\mathrm{output}\;\mathrm{rate}\;\left(\frac{g}{\min}\right)=\frac{\mathrm{Weight}\;\mathrm{of}\;\mathrm{nebulized}\;\mathrm{liquid}\;\left(g\right)}{\mathrm{Nebulization}\;\mathrm{time}\;\left(\min\right)}$$

### 2.7. Sterilization of NE Formulations

TD-loaded NEs were sterilized using two techniques: autoclave (steam sterilization) using a portable Gallenkamp autoclave at 121 °C for 15 min and filtration through 0.22 µm cellulose acetate syringe filter (Minisart^®^, Sartorius, Goettingen, Germany). The stability of NEs upon sterilization by each technique was evaluated in terms of the percentage of drug remaining and globule size after 50-fold dilution with filtered distilled water.

### 2.8. In-Vitro Cytotoxicity Assessment Using MTT Assay

Biocompatibility of the formulations was tested on the A549 cell line using an MTT assay. Cells were propagated in a humidified air atmosphere (37 °C, 95% relative humidity (RH), and 5% CO_2_) in DMEM that was fortified with 10% FBS, 1% (*v*/*v*) L-glutamine and 1 g/L glucose. Then, the cells were seeded into 96-well microtiter plates at a cell density of 5 × 10^3^ cells/well and incubated for 24 h. Afterward, the media were replaced by 100 µL of fresh DMEM, where in the case of the negative control wells, only DMEM was added, while in the other wells, the cells were exposed to various concentrations of previously sterilized TD-loaded NE formulations after adequate dilution with DMEM. After 3 h of incubation, the media were replaced by fresh DMEM, and cells were left to grow overnight in the incubator. On the next day, a stock solution of 5 mg/mL of MTT solution (in PBS) was prepared, diluted to 1 mg/mL with serum-free culture medium, and a 120 µL was added to each well after removal of the old medium. Following incubation for 3 h, the medium was removed by slow aspiration and replaced by 100 µL of dimethyl sulfoxide (DMSO). Then the plates were incubated for 5 min at 37°C followed by shaking for 10 min to dissolve the formed purple-colored formazan crystals. Absorbance was measured at 570 nm using xMarkTM Microplate Absorbance Spectrophotometer (Smart Spec, Bio-Rad Laboratories, Hercules, CA, USA). The viability of the NE-treated cells was expressed as a percentage relative to the control, according to the following equation:

% Cell viability = ([Abs] test)/([Abs] control) × 100%.

where ([Abs] test) is the absorbance of each concentration of the test substance, ([Abs] control) is the absorbance obtained from the untreated cells. The last reading was supposed to correspond to 100% cell viability. The results are presented as the means ± standard deviation (S.D.) of triplicate samples.

### 2.9. In-Vivo Lung Toxicity Evaluation

#### 2.9.1. Experimental Animals

Male Sprague-Dawley rats (average weight 200 g) were used for the in-vivo toxicity study. Animals were housed in the animal house of the faculty of medicine (Alexandria University, Alexandria, Egypt) under controlled temperature (21 ± 1 °C), humidity (65% RH), and lighting (10 h light/14 h dark cycle). Two weeks were given for the animals to adapt to their environment before commencing the experiment. Standard chow and water ad libitum were allowed throughout the study duration. The study protocol was approved by the ethical committee of Alexandria University. Experimental procedures were conducted according to the ethical guidelines of Alexandria University and the European Council Law for animal care (EEC Directive of 1986, 86/609/EEC) to ensure minimal animal suffering.

#### 2.9.2. Animal Dose Calculation

Two steps were followed to determine the proper dose of TD for treating PAH in rats following oro-tracheal administration. The first step was the conversion of human pediatric oral dose to rat oral dose. Unfortunately, no dose of TD has been approved by the FDA for treating pediatric PAH. However, a study reported that a quarter or half of the adult dose was safe and effective for treating pediatric patients [33]. Thus, half the adult dose (20 mg) was selected in the present study as the pediatric oral dose and it was extrapolated to the rat oral dose based on the standard table of Paget and Barnes [34]. The second calculation step was the conversion of the oral dose to the pulmonary dose. Pulmonary administration was reported to decrease the required dose by 40–100 times compared to the oral route [30]. Based on this information, a rat weighing 200 g should administer a pulmonary dose of TD ranging between 3.6 µg to 9 µg.

#### 2.9.3. Study Design

The safety of the selected formulations was evaluated following oro-tracheal delivery into the rat lungs through biochemical analysis of bronchoalveolar lavage (BAL) and histopathological examination of lung slices. On the day of the experiment, animals were randomly assigned into five groups (*n* = 5). Animals of one of the groups were administered saline as a negative control. On the other hand, those in the other groups administered the two selected formulations at the two dosing levels after appropriate dilution with saline as follows: C4_TD(3.6)_ group [C4_TD_ (3.6 µg)], C4_TD(9)_ group [C4_TD_ (9 µg)], P2_TD(3.6)_ group [P2_TD_ (3.6 µg)] and P2_TD(9)_ group [P2_TD_ (9 µg)].

#### 2.9.4. Oro-Tracheal Administration

Prior to the procedure, animals were intraperitoneally anesthetized with a cocktail of ketamine (80 mg/kg) and xylazine (10 mg/kg). Animals of the control group were administered 100 µL saline through the oro-tracheal route. By adopting the same technique, animals of the other groups were administered 100 µL of the diluted formulations, which included the calculated drug doses. Rats were held tightly at a 45° angle. For ultimate oropharyngeal exposure, the mouth was kept open by blunt forceps and another was used to help displace the tongue. A light source was also adjusted for optimal illumination of the rat’s trachea. When the trachea became visible, a syringe equipped with a 22-gauge intravenous catheter was introduced into the trachea after pushing it against the soft palate. Afterward, the catheter was inserted approximately at the bottom of the trachea when the tracheal cartilage ring was sensed, and the liquid sample was injected gently. Then, the catheter was slowly removed, and the rat was held vertically for one min to prevent reflux or regurgitation and to allow the administered fluid to distribute in the lungs. The animals were euthanized via ether inhalation 24 h post-oro-tracheal administration, BAL was performed, and the lungs were collected for histopathological examination.

#### 2.9.5. Biochemical Assessment of BAL Fluid

BAL was performed by injecting and withdrawing 4 mL aliquot of sterile saline into the lungs through the trachea three consecutive times. The collected BAL fluid was centrifuged for 5 min, 400 rpm, at 25 °C. The supernatant was analyzed for lactate dehydrogenase activity (LDH) and total protein content using commercial kits (Roche Diagnostics. Indianapolis, IN, USA). Statistical analysis was carried out using an unpaired student’s *t*-test. The significance level was set at *p* < 0.05.

#### 2.9.6. Histopathological Examination

After completion of lung lavage, the lungs were washed with saline and preserved in a 10% formalin solution, followed by washing and dehydration using alcohol. Samples were then cleared in xylene, embedded in paraffin at 56 °C, and dried in a hot air oven for 24 h. This was followed by the sectioning of paraffin tissue blocks at 4 µm by sledge microtome. The specimens were then deparaffinized and stained by hematoxylin and eosin (H & E) stains for histopathological inspection using light microscopy.

### 2.10. Long-Term Stability Study

A long-term stability study was performed for three months at different storage conditions, RT, refrigerator (2–8 °C), and 40 °C/75% RH. Samples were evaluated at 0, 1, 2, and 3 months for signs of physical instability (phase separation or drug precipitation) through visual examination. In addition, globule size, %T and drug content were determined.

## 3. Results and Discussion

### 3.1. Screening of SAAs and Co-SAAs: Evaluation of Dispersion Properties

The current work endeavors to prepare oil-in-water (o/w) NE formulations of TD that can resist aqueous dilution to be administered via nebulization. Upon dilution of lipid/SAA mixtures, different systems can be obtained depending on the composition and the concentration of the employed ingredients [35]. Dilutable NE concentrates are scarce, as most oil-based formulation concentrates are destroyed upon aqueous dilution accompanied by migration of the solubilized drug to the outer continuous phase, followed by precipitation [24]. No systematic approach has been described in the literature for selecting lipids for a certain dosage form [35]. Many marketed lipid-based drug products appear to result from trial-and-error experiments [35]. Therefore, a detailed pre-formulation study was undertaken to select the appropriate excipients for formulating a dilutable NE formulation of TD.

#### 3.1.1. Effect of Oily Phase

In the present study, Capmul-MCM-EP was selected as the oily phase since it was the oil in which TD exhibited the highest solubility, as manifested by our research group [17]. A study by Prajapati et al. showed that this oil, which is mainly a monoglyceride, produced both NE and emulsion phases when mixed with Cremophor EL and water [35]. However, an interesting finding was the expansion of the NE region when Capmul (monoglyceride) was mixed with Captex 355 (triglyceride) in a (1:1) weight ratio. In addition, it was shown that the dispersion of a model drug in an aqueous medium of the oil mixture was superior to that when using individual lipids [35]. Thus, in the present study, Labrafac-lipophile WL 1349 (a medium chain triglyceride of (50 to 80%) caprylic acid (C_8_) and (20 to 50%) capric acid (C_10_)) were added to Capmul in an equal weight ratio to improve the ability of the latter to be nano-emulsified.

The oil mixture (Capmul/Labrafac (1:1) *w*/*w*) was blended with various SAAs followed by aqueous dilution, then, the efficacy of different SAAs to nano-emulsify the oily phase was evaluated by visual assessment of the resultant mixtures as demonstrated in Table 1. Capmul and Labrafac were included as single oils in the study for comparison. As shown in Table 1, when the oil mixture was employed as the oily phase, the formulations were well nano-emulsified with all employed SAAs except Labrasol and Poloxamer-407. However, Labrasol exhibited the highest solubility for TD among other studied SAAs, as previously demonstrated by our research group [17]. Thus, to maintain TD solubility in the formulation, different surfactant mixtures (S_mixs_) were prepared in which Labrasol was blended with other SAAs in a (1:1) weight ratio, and their nano-emulsifying efficacy was studied (Table 1).

As shown in Table 1, a clear NE was formed in the case of the oil mixture with all the studied S_mixs_ except the S_mix_, in which Tween-80 was included. Conversely, single oils showed difficulty in being nano-emulsified. Both oils could be nano-emulsified with some of the employed SAAs, but NE could not be produced with any of the studied S_mixs_. The findings of the present study highlighted the superiority of the oil mixture, as it was easier to be nano-emulsified by various S_mixs_, unlike the situation for individual oils. These results were in good agreement with those reported by Prajapati et al., in which the oil mixture of monoglyceride and triglyceride prepared in a (1:1) weight ratio was found to be the optimum oil among the other oily phases studied as it could produce the widest NE region and the size of the produced NEs was smaller than that obtained from the individual monoglyceride and triglyceride. Thus mixing the monoglyceride with the triglyceride in (1:1) weight ratio had the optimum impact in promoting the NE formation [35].

The minimal ability of Capmul solely to form a NE is attributed to its polarity as it has an HLB value of 4.7 [35]; it exhibits certain solubility in water, and as a result, Ostwald ripening takes place; consequently, emulsions are produced rather than NEs. Ostwald ripening could be inhibited through the incorporation of a second oil of high molecular weight and of much lower continuous phase solubility, such as medium- or long-chain triglycerides, defined as ‘ripening inhibitors’ [36,37,38]. This additional oil retards Ostwald ripening through an entropy of mixing effect, which counteracts the imbalance of droplet size effect [38]. Labrafac is a medium-chain triglyceride possessing an HLB value of 1 [39]; its poor aqueous solubility acted as a kinetic barrier to Ostwald ripening. Consequently, stable NEs were produced when mixed in an equal weight ratio with Capmul. Several works were reported in the literature in which a ripening inhibitor oil was mixed with the relatively polar oil to reduce its aqueous solubility, inhibit Ostwald ripening and ensure the system’s long-term stability [36,37].

#### 3.1.2. Effect of SAAs

- Single SAAs

The study was undertaken to identify the SAA with the highest emulsification efficiency. The oil-to-SAA ratio was established according to the basis of requisites reported by Pouton for spontaneously emulsifying systems [40]. Concerning the selection of SAAs and Co-SAAs, drug solubility would come beyond the major selection perspective, emulsifying efficiency, as the TD dose that is supposed to be used in treating pediatric PAH via inhalation is small, as will be discussed later. Safety is a major determining factor in choosing an SAA. All the SAAs screened in the current study belong to the non-ionic SAAs class and are less toxic than their ionic counterparts [40]. An additional important criterion for the selection of SAAs is the HLB value. The HLB value required to form o/w NEs is greater than 10 [40,41]. It was reported that the high HLB value of SAAs facilitates the instantaneous formation of o/w droplets and permits rapid formulation spreading in aqueous media [42]. The HLB values of all the employed SAAs are higher than 10, thus fulfilling this requirement.

Self-emulsification is influenced by many factors, such as the nature of the oil/SAA combination, the concentration of SAA, the ratio of oil to SAA, and the temperature at which self-emulsification takes place [42]. Only limited combinations of pharmaceutical excipients could produce effective self-emulsifying systems [42]. As presented in Table 1, both Cremophor EL and Cremophor RH40 exhibited superior nano-emulsifying capacity among the screened SAAs, as they could nano-emulsify the three oily phases, followed by Tween-80 that could nano-emulsify the oil mixture and Labrafac, then Poloxamer-407 which could only nano-emulsify Capmul. Whereas, Labrasol appeared to be a poor nano-emulsifier as it could not nano-emulsify any of the oily phases employed. Although the HLB values of the SAAs employed in the current study were quite similar, in the range of 12 to 16, except for Poloxamer-407, there was a great difference in their nano-emulsification ability; this confirms that the SAA HLB is not the sole parameter determining the SAA’s ability to form NE. This observation was in line with the investigations reported by Warisnoicharoen et al., who concluded that nano-emulsification is also influenced by the structure and chain length of the SAA [43]. Furthermore, Nepal et al. also reported that multiple variables other than the HLB value significantly affect the emulsification process and its efficiency, including the lipid-SAA affinity and the visco-elasticity of the system [41].

It was reported that the SAA alkyl chain structure affects the oil penetration onto the curved film of SAA hence resulting in the formation of self-nano-emulsion [44]. The obtained findings coincided with those reported by Rao et al., in which Cremophor EL and Cremophor RH40 exhibited the highest emulsification efficiency with Lauroglycol FCC and Caproyl-90, followed by Tween-80 and neither Tween-20 nor Labrasol provided any self-nanoemulsifying drug delivery system (SNEDDS) prototype [44]. Capryol-90 is propylene glycol mono caprylate, i.e., monoester of caprylic acid (C_8_) [40], and its structure is closely related to Capmul and Labrafac. In this study, Cremophors were shown to be superior to Tween-80 in their ability to form a self-nano-emulsion; this was attributed to the fact that Cremophors have branched alkyl structure, whereas, Tween-80 has a linear chain alkyl structure [44]. It was reported that the branched alkyl structure of SAAs was more favorable for self-NE formation [45].

Concerning Labrasol, a study by Zhang et al. proved that Labrasol was a less efficient emulsifier than Cremopher RH40 and Cremopher EL because the self-nano-emulsifying regions provided by Cremophors were observed to be larger [46]. Another study by Kassem et al. also demonstrated the poor emulsifying ability of Labrasol with lemon oil compared to Cremophor EL and Tween-80 [45]. The poor nano-emulsifying properties of Labrasol could be ascribed to its poor affinity to the oils employed in the current study, which reduced its adsorption onto the droplets [45]. In light of the aforementioned results, it can be concluded that Labrasol can only be convenient for developing a self-emulsifying drug delivery system (SEDDS) of TD and not for SNEDDS with respect to Capmul/Labrafac (1:1) (*w*/*w*) oil mixture.

- S_mixs_

Labrasol attempts to form a NE with the oil mixture were unsuccessful; nevertheless, its (1:1) (*w*/*w*) mixtures with other SAAs were screened in order to conserve TD solubility. As demonstrated in Table 1, Labrasol could yield a NE with the oil mixture in the presence of Cremophor EL, Cremophor RH40, and Poloxamer, while in the case of Tween-80, NE formation was not feasible. These findings inferred that both Cremophor EL and Cremophor RH40 could enhance the efficiency of Labrasol to solubilize the oil mixture and water into a single-phase transparent system, while Tween-80 could not.

The generated data were quite consistent with those reported by Djekic et al., in which isopropyl myristate preconcentrates incorporating Cremophor RH40 as a single Co-SAA afforded the largest range of water solubilization upon mixing it in a 1:1 weight ratio with Labrasol, followed by Solubilisant gamma^®^ 2429 (octoxynol-12 + Tween-20 + PEG-40 hydrogenated castor oil), whereas Solubilisant gamma^®^ 2421 (octoxynol-12 + Tween-20) exhibited the least water incorporation capacity [47]. Furthermore, the results of photon correlation spectroscopy (PCS) revealed NE with monomodal size distribution and low PdI (<0.25) upon using Cremophor RH40 as a sole Co-SAA. On the other hand, NEs of bimodal size distribution and PdI > 0.2 were formed in the case of Solubilisant gamma^®^ 2421 and Solubilisant gamma^®^ 2429, reflecting heterogeneity in the globule size [47]. These results were attributed to the role of Cremophor RH40 for interface flexibility. On the contrary, the excessive addition of water forces the more hydrophilic components (octoxynol-12 and Tween-20) to migrate out of the interface to a greater extent than that of Cremophor RH40, reducing their overall concentration at the interface, thus destabilizing the NE. Based on the aforementioned data, the results of our investigation, alongside that of the Djekic et al. study, assert the superiority of Cremophors in Labrasol-based NEs over Tweens, as the interfacial film of their resultant NEs is less sensitive to aqueous dilution.

Since Labrasol blends with both Cremophor RH40 and Cremophor EL had comparable nano-emulsifying behavior, a (1:1) (*w*/*w*) mixture of Labrasol with Cremophor EL was selected for further studies because TD solubility in Cremophor EL was higher, as previously reported by our research group [17].

Despite the use of neither Labrasol nor Poloxamer alone resulting in NE formation when mixed with the oil mixture (Table 1), the use of the S_mix_ could yield a NE (Table 1). It was previously reported that mixing Labrasol with Poloxamer-407 in (80:20) and (60:40) weight ratios resulted in clear colloidal systems after dilution with water [48]. Poloxamers are triblock copolymers; their structure is based on a central hydrophobic polyoxypropylene (POP) portion connected to hydrophilic polyoxyethylene (POE) chains [49]. Poloxamers are categorized as steric SAAs; they stabilize the droplets through steric repulsion. Steric SAAs have bulky groups projecting into the continuous phase, thus creating a brush-like barrier surrounding the droplets. An additional advantage that Poloxamers offer to NEs is hindering the destabilization caused by Ostwald ripening; this is due to the strong adsorption of Poloxamers at the oil-water interface due to their lower aqueous solubility, which makes them desorb with difficulty from the interface during the ripening phenomenon, decreasing the rate of instability occurrence significantly [37,49]. Furthermore, adding a polymeric SAA to the non-ionic SAA enhances the emulsifying process, where the polymeric SAA influences a critical parameter involved in the emulsifying process, ‘the interfacial dilational modulus’ [37]. Thus, a S_mix_ of polymeric and non-ionic SAAs would be optimal for promoting an efficacious emulsifying process via increasing the dilational surface modulus and decreasing the interfacial tension of the shrinking drops [37].

The aforementioned results suggested the use of a S_mix_ of Labrasol with either Cremophor EL or Poloxamer-407 for further investigations.

#### 3.1.3. Effect of Co-SAAs

It was reported that the dispersibility of a drug and its absorption from the formulation could be enhanced by adding a Co-SAA to an SAA-containing formulation [50]. Thus, different Co-SAAs were screened for their ability to nano-emulsify the oil mixture after blending them with a (1:1) (*w*/*w*) S_mix_ of Labrasol and Cremophor EL, which was used as an SAA model. The Co-SAAs employed in the present study were: Transcutol HP, PEG 400, propylene glycol, and ethanol. In spite of the fact that Co-SAAs were included in the preparation of SNEDDS in the literature [50], unfortunately, their addition in the current study resulted in the formation of an emulsion rather than a NE. These results were in good agreement with those reported by Shakeel et al., in which Labrasol could not form a NE with Caproyl-90 in the presence of Co-SAAs of lower HLB values at the investigated composition, and it was stated that Co-SAAs of higher HLB values could probably offer a suitable alternative [51].

The addition of a Co-SAA during NE fabrication has been linked to many problems. First, it increases the risk of NE destabilization [52]. As it was reported that the efficacy of the SAA/Co-SAA mixtures to maintain the interface integrity during aqueous dilution decreases by increasing the relative Co-SAA content [52]. This is because, in o/w NE systems, the aqueous solubility of Co-SAAs is often greater than that of the primary SAA. Consequently, upon diluting the o/w NE, the Co-SAA partitions to the aqueous phase more strongly, diminishing the Co-SAA concentration at the interface and destabilizing the NE droplet [53]. Another obstacle that confronts the lipid systems containing these components is their susceptibility to drug precipitation upon dilution, unlike in the presence of hydrophilic SAAs. SAAs are less likely to lose their solvent capacity upon dilution with water because the relationship between the drug solubility and the concentration of the hydrophilic SAAs is linear above the critical micelle concentration (cmc), whereas it approximates a logarithmic relationship with the Co-SAA concentration [54].

Given the above-mentioned undesirable effects of Co-SAAs, Co-SAA-free o/w NEs were formulated. The combined use of SAAs is an alternative approach that can be adopted instead and has gained much attention in the literature [40,55,56]. A study carried out by Borhade et al. in which the authors concluded that the use of a S_mix_ was the optimum selection compromise as it provided many privileges over using an SAA/Co-SAA mixture, including the widening of the NE region and the broadening of the oil compositions so that high drug loading can be achieved [40].

Based on the results of preliminary screening, in order to achieve improved physical stability, it was recommended to formulate NEs for further investigations using a mixture of Capmul and Labrafac (1:1) (*w*/*w*) as the oily phase and a S_mix_ of Labrasol with either Cremophor EL or Poloxamer-407.

### 3.2. Construction of Pseudo-Ternary Phase Diagrams

**Figure 1:**
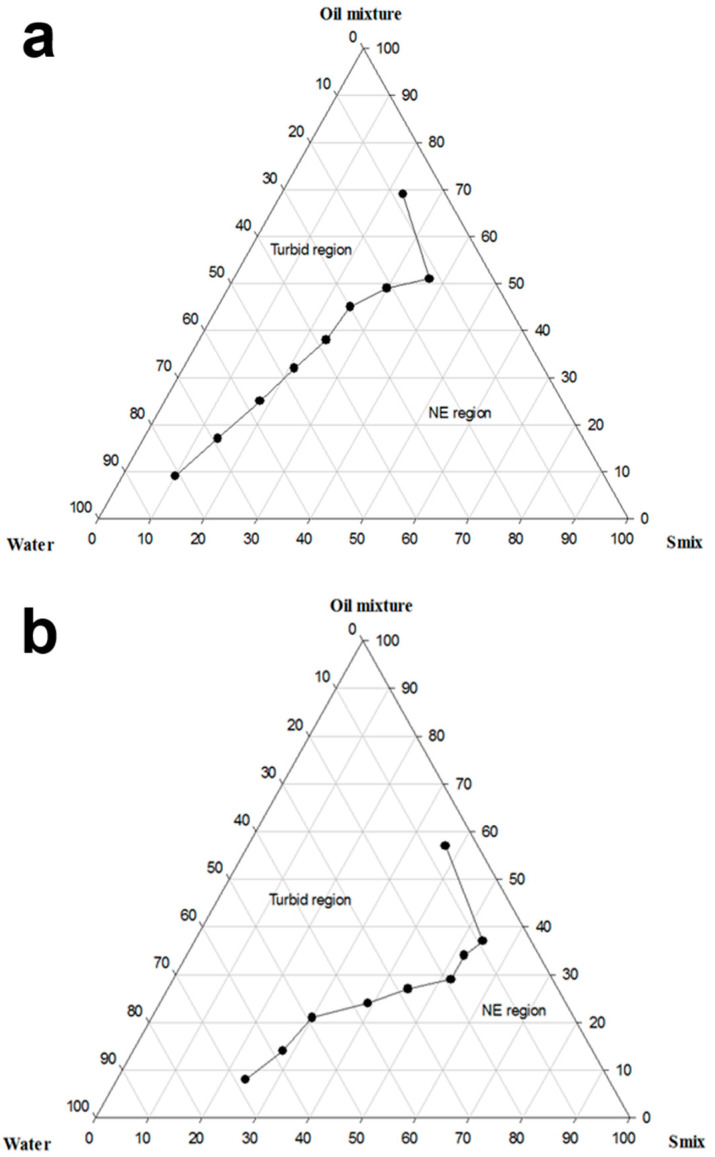
Pseudo-ternary phase diagram of (**a**) system B (Capmul-MCM-EP:Labrafac-lipophile WL 1349 (1:1) (*w*/*w*)-Labrasol:Cremophor EL (1:1) (*w*/*w*)-Water) and (**b**) system C (Capmul-MCM-EP:Labrafac-lipophile WL 1349 (1:1) (*w*/*w*)-Labrasol:Poloxamer-407 (2:1) (*w*/*w*)-Water).

Phase diagrams were constructed to show the possible concentration of components that can yield a NE. Phase diagrams of two different systems (B and C) were constructed according to the results of the preliminary investigations (Figure 1).

Utilizing a binary mixture of non-ionic SAAs has a synergistic effect on the HLB, which boosts the SAA layer’s flexibility at the interface and increases the SAA partitioning at the oil-water interface, thereby stabilizing the o/w NE [40]. As demonstrated in Figure 1a, system B yielded a NE containing as high as 50% oily phase and 70% aqueous phase. On the other hand, as shown in Figure 1b, system C could produce a NE having a maximum oil concentration of 30%, and the amount of water that could be nano-emulsified reached 60%. %T of the formulations used to construct system B varied between 95.4 ± 0.2% and 100 ± 0.1%, whereas it ranged between 96% to 99.2 ± 0.1% in the case of system C. System C containing Poloxamer-407 exhibited a narrower NE region than that provided by system B that includes Cremophor EL; this might be attributed to the higher amount of Labrasol employed in system C, which resulted in a lower nano-emulsification ability. Upon comparing system B with system A, previously constructed by our research group, the broadening of the NE region is observed upon employing the oil mixture as the oily phase and utilizing S_mix_ for nano-emulsification instead of the SAA/Co-SAA mixture. This is ascribed to employing the oil mixture that inhibited Ostwald ripening, removal of the Co-SAA that partitions away from the interface upon aqueous dilution, and utilizing a S_mix_ of an appropriate HLB facilitating the nano-emulsification process.

In conclusion, systems B and C were considered for further investigations that encompassed comparing between formulations of the two systems regarding the physicochemical properties, stability, suitability for the pulmonary application, and safety.

### 3.3. Selection Strategy and Preparation of Placebo NEs

Different formulae of varying compositions were selected from both systems for drug incorporation and formula optimization. The detailed composition of the formulations is presented in Table 2. All NE compositions were presented as (%*w*/*w*). The formulations were selected to contain a fixed amount of oily phase (10%), and the amount of SAAs used was varied at the expense of water to produce formulations of variable SAA concentrations. In order to study the effect of the amount of oily phase, formulations C4 and P2 were selected to have the same amount of SAAs (40%) as the formulations C3 and P1, respectively, but a lower concentration of oily phase (5%) was incorporated.

An optimum NE formulation for pulmonary delivery of TD was selected based on the following criteria:

- It should be able to solubilize the required dose of the drug completely.
- It should be stable upon aqueous dilution without phase separation or drug precipitation.
- It should contain the least possible concentration of SAAs to avoid irritation upon use.

Based on the above criteria, various tests were carried out to aid in selecting the optimum formulation. NEs were first exposed to 50-fold aqueous dilution in order to simulate the administration conditions, as will be discussed later, and the stability of the diluted formulae was evaluated. The physical appearance of the diluted formulations after 2-h storage at RT is presented in Table 2.

As depicted in Table 2, all formulations stayed clear and showed no signs of physical change (cloudiness or phase separation) after dilution except the formulations P1 and C5. P1 had the same amount of SAAs (40%) as formulation P2, but the oil content of P1 was 5% higher. The turbidity observed with P1 might be attributed to the higher quantity of oil, as the amount of SAAs in the formulation was inadequate to nano-emulsify the oil with water. Regarding C5, this formulation had the same oil content as the formulae C1, C2, and C3, but the amount of SAAs varied at the expense of water. So, the reason C5 could not retain its physical integrity upon dilution may be ascribed to the low SAA content, as C5 included the least amount of SAAs (30%) among the inspected formulations. This amount of SAAs was not enough to retain the stability of the NE droplets after dilution, and an emulsion was formed instead. Based on the previous results, formulations C3, C4, and P2 were selected for further studies, as they could maintain their physical stability upon aqueous dilution while accommodating the least possible amounts of SAAs.

### 3.4. Preparation of TD-Loaded NE Formulations

#### 3.4.1. Drug Loading

In order to achieve a satisfactory drug loading, it is important that the entire therapeutic drug dose be soluble in a reasonable and small volume of the formulation. If the drug solubility in the formulation were insufficient, there would be a chance for the drug to precipitate upon aqueous dilution [8]. Therefore, a solubility study was performed to determine the maximum drug loading of each formulation; results are presented in Table 2. It was observed from the results that the maximum drug loading of the investigated formulations was not high, but all of them were more than 2 mg/mL; this drug loading was sufficient for the preparation of TD formulations for our aimed purpose. The low solubility of TD in the investigated NEs was attributed to the hydrophilicity of SAAs, as the HLB values of the employed SAAs are high. Similar findings were reported by Wang et al. in which the solubility of nalbuphine prodrugs in Brij 98-containing submicron lipid emulsion was low. This phenomenon was attributed to two reasons. The first reason was the large hydrophilic parts of the employed co-emulsifier (Brij 98, HLB = 15.3), which made it difficult for the lipophilic prodrugs to interact with amphiphiles possessing limited lipophilicity. The second explanation was the flexibility of the SAA film, which resulted in the leakage of drug molecules from the inner phase into the outer phase [57].

It was noteworthy from Table 2 that TD solubility in C4 was higher than that in the corresponding formulation containing Poloxamer-407 (P2); this was attributed to the low solubility of TD in Polaxamer 407 when compared with that in Cremophor EL (10.305 mg/mL at 30 °C) [17]. It was reported that the solubility of TD in 20% (*w*/*v*) aqueous solution of Poloxamer-407 at 37 °C was 0.5 mg/mL. In addition to the drug solubility in the investigated SAAs, many other parameters affect the SAA’s drug solubilization capacity, including the SAA structure, cmc, and HLB value [58]. As TD is a hydrophobic drug, it will prefer to reside at the hydrophobic interface; consequently, its solubility will be higher in the presence of SAAs of lower HLB value. Since Cremophor EL possesses a lower HLB value (12–14) than Poloxamer-407 (18–23), it was able to solubilize more of the hydrophobic drug. Similar results were reported in the literature by Kogan et al. [58].

#### 3.4.2. Formulation Dispersion–Drug Precipitation Test

The formulated NEs are supposed to be diluted before nebulization to be used for drug delivery into the lungs. Upon dilution of the NEs, precipitation is likely to occur if the concentration of the drug in the formulation exceeds the equilibrium solubilization capacity of the system [59]. It was reported that the reduction in solubilization capacity was attributed to an alteration in the locus of drug solubilization associated with the microstructural transitions of the system during aqueous dilution [60].

This test was carried out to predict whether precipitation was likely to occur upon aqueous dilution. Since the prepared formulations were highly concentrated relative to the therapeutic dose (25 μg), it was supposed to approximately dilute the formulations 300 folds to nebulize a four-milliliter solution. Nevertheless, a 50-fold dilution was chosen in the current investigation as an example of a high dilution ratio. The results of the study are presented in Table 2. As demonstrated in the table, all formulations could retain more than 95% of the initial drug content by the end of the experiment. These findings confirmed that the formulations could preserve the dissolved drug within the system after aqueous dilution for a reasonable period of time.

Based on the solubility study and the dispersion tests, the selected formulations C3_TD_, C4_TD_, and P2_TD_ proved to be the optimal selection compromise as they could fulfill the conditions of the selection criteria. These formulations were able to dissolve the required dose of the drug, they could be subjected to aqueous dilution without phase separation or considerable drug crystallization, and they included the least possible amount of SAAs.

### 3.5. Physicochemical Characterization of Selected NE Formulations

Upon examining the placebo NEs using a cross-polarized light microscope, the field of view remained dark, thus confirming the homogeneity and the optical isotropy of the prepared systems [17].

Refractive indices of the placebo and TD-loaded NEs ranged between 1.343 and 1.348, as presented in Table 3. These values were very close to water 1.33; this inferred that the investigated NEs were of o/w type. Values of the refractive index of the drug-loaded formulations were very close to that of the corresponding placebo formulations. These findings confirmed that the NEs were isotropic and likely had no interactions between the drug and the NE components [61]. Values of %T of the investigated placebo NEs and their corresponding TD-loaded formulations were close to 100%, as depicted in Table 3, thus indicating the clarity of the formulations and the presence of the oil droplets in the nanometer range [61].

The tonicity and the pH value of the aerosolized solutions are very critical parameters that have to be controlled; otherwise, aerosol-induced coughs will be observed [25]. For this reason, NEs in the current study were diluted using an isotonic saline solution to adjust the osmolarity of the formulations. In addition, the pH values of the saline-diluted formulations were measured to ensure their suitability for inhalation since the lungs possess limited buffering capacity [25]. Table 3 shows that the pH values of the diluted formulations lie within the range recommended by the European pharmacopeia for nebulized liquid preparations (3–8.5) [25]; hence these formulations were suitable for the pulmonary application.

Results of viscosity measurement (Table 3) demonstrated that all the diluted formulations had a low viscosity despite the NEs included ingredients of high viscosity; these components had no pronounced effect on the measured viscosities due to the high fold of dilution applied to the formulations. It was also noticed that the viscosity of the placebo and TD-loaded formulations were almost similar; this meant that the drug had no influence on the viscosity. It was reported that jet nebulization is more effective with regard to the respirable output in the case of liquids possessing low viscosity (1–6 cP) [62]. The viscosity of the saline-diluted TD-NEs was within the recommended range except for C4_TD_, which possessed a slightly higher viscosity (7.5 cP) that might not considerably affect the nebulizer performance since it was reported in a study carried out by McCallion et al. that jet nebulizers could generate aerosol with an optimal respirable output (high output and low mass median diameter) from median-viscosity liquids and they could even nebulize 20–40% of the most viscous silicon fluid studied (97 cP) [63]. In summary, the low viscosity values of the current investigation suggested the feasibility of nebulizing the saline-diluted formulations using a jet nebulizer. As nebulization of highly viscous fluids results in a small mass median diameter, long nebulization time, and low output rates [63].

Zeta potential values of saline-diluted placebo and TD-loaded NEs were close to neutral ranging from −1.42 to 1.15 mV (Table 3), as the SAAs employed in the formulations were non-ionic [17]. Similar findings were reported by Nesamony et al. [8,9]. Neutrally-charged NEs were reported to permeate successfully through different biological membranes [8]. Moreover, it was reported that using non-ionic SAA-based NEs could improve the bioavailability in different animal models without causing toxicity [9]. Concerning the stability of NEs with respect to the charge, NEs with neutral zeta potential was reported to possess sufficient stability [29].

The globule size was measured for the placebo and drug-loaded formulations after 50-fold dilution with water. Globule size of concentrated NEs could not be determined because of micellar scattering. In addition, globule size was also measured after dilution of TD-NEs with saline, as the presence of salts may affect the stability of the formulations. Table 4 summarizes the globule size and PdI of the diluted formulations. The results demonstrated that the mean globule size of placebo NEs were less than 100 nm with a narrow PdI value (<0.2). It was also shown that the incorporation of TD had no considerable effect on the size of NE droplets. Moreover, it was observed that upon dilution of TD-NEs with saline, the globule size stayed approximately the same, confirming the stability of the formulations in the presence of saline. Similar results were reported by Nesamony et al. [8,9]. The small size of NE droplets (less than 100 nm) was reported to improve the rate and extent of drug absorption. Furthermore, this small size allows the quick breakdown of oil releasing the drug and makes the formulation suitable for delivery via different routes, including the pulmonary route [8,9].

Results of globule size analysis also showed that the globule size of C4_TD_ was smaller than that of C3_TD_; this is attributed to the higher SAA:oil ratio in the case of C4_TD_, which increased the amount of SAA molecules at the interface [64]. In other words, increasing the oil content in the case of C3_TD_ will increase the number of oil droplets, requiring a higher amount of SAA to keep the globule size constant [64]. However, since the amount of SAA was fixed at 40% for both NEs, a reduction of the interfacial film density of the SAA will occur in the case of C3_TD_. To compensate for this, merging of some oil droplets into bigger ones will take place, increasing the globule size [64]. Furthermore, it was also observed that the mean globule size of P2_TD_ was higher than that of the corresponding NE, C4_TD_. In addition to the higher amount of Labrasol utilized in P2_TD_, which has influenced the emulsification efficiency and the globule size, the different interfacial behavior of the different SAAs employed in the two formulations (Cremophor EL and Poloxamer-407) might have contributed to the difference in globule size [65]. It was reported that small molecule SAAs, like Cremophor EL in the current study, can be adsorbed easily onto the droplet surface, resulting in a reduction in the interfacial tension and formation of small droplets [65]. On the other hand, the large molecular structure of SAAs, as in the case of Poloxamer-407 in the present study, precludes close packing of the points of contact with the interface; as a result, less reduction in the interfacial tension occurs [65].

**Figure 2:**
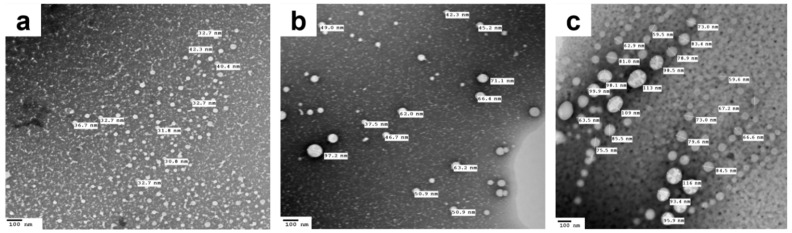
TEM images showing oil droplets of (**a**) C3_TD_, (**b**) C4_TD_ and (**c**) P2_TD_ NEs after 50-fold dilution with saline.

TEM images of the saline-diluted TD-NEs (Figure 2) revealed spherical-shaped globules of homogenous size distribution within the nanometer range and possessing no micellar aggregates. Furthermore, it was noticed upon measuring the size of the nanodroplets using TEM that it was larger than those obtained from the dynamic light scattering (DLS) experiments. For example, the globule size of C3_TD_ determined by DLS after dilution with saline was 18.77 nm ± 0.08, whereas the size discerned using TEM for the same formulation ranged from 30.8 to 42.3 nm. This divergence was ascribed to the variation in the experimental conditions utilized in the two techniques [8,9].

The thermodynamic stability of TD-loaded NEs was studied via accelerated stability tests, including centrifugation and freeze-thaw cycle stress tests. Upon visual examination, all the formulations demonstrated no signs of phase separation or drug precipitation after both stress conditions. In addition to the visual assessment, the colloidal stability of the formulations was also evaluated following these tests (Table 5). Results showed that the globule size of the formulations stayed approximately the same following centrifugation. However, limited aggregation (4793 nm ± 164.8, 3.2% ± 0.7) was detected in the case of P2_TD_ after centrifugation, and a slight change in the globule size after freeze-thaw cycles were observed for C3_TD_ (33.19 nm ± 1.18) and P2_TD_ (44.56 nm ± 0.9) when compared with the initial size, (18.66 nm ± 0.05) for C3_TD_ and (25.74 nm ± 0.05) for P2_TD_. Whereas the globule size of C4_TD_ remained constant at 15 nm after both tests. Despite the change in the size of the two formulations, C3_TD_ and P2_TD_, their size was still less than 100 nm with a PdI value of 0.2 or less. Thus, it can be concluded that there was no apparent change in the globule size and PdI, indicating the thermodynamic stability of these systems. The stability of the NEs fabricated in the current study is attributed to their small globule size, which allows the droplets to undergo Brownian motion sufficient to overcome the influence of the stress conditions [11]. C4_TD_ demonstrated the highest stability among the studied NEs as it has the smallest globule size, which in turn decreases the coalescence rate and increases the thermodynamic stability of the system [11].

### 3.6. Nebulization Performance of TD-Loaded NEs

In the current work, targeted delivery of TD to the lungs via inhalation was chosen for the treatment of pediatric PAH since it is believed that inhalation therapy offers many privileges over oral delivery in case of respiratory diseases, as previously mentioned. Three categories of inhalation devices are available, among which nebulizers were the first developed devices for pulmonary application [25]. Nebulizers possess many merits, including being advantageous in case of diseases requiring high pulmonary doses, and they are useful for patients who cannot achieve sufficient coordination necessary during the use of other devices (e.g., pediatric patients) [66]. For the latter reason, nebulizers were selected in the current investigation.

Nebulizers act through the aerosolization of drug solutions. In order to guarantee therapeutic comfort, two substantial parameters, aerosol output, and aerosol output rate were taken into consideration [10]. Results showed that the aerosol output varied between 94.4 and 96.8% for the three formulations (Table 6). The amount of fluid delivered by the nebulizer is referred to as ‘aerosol output’ [67]. It is important that the aerosol output is high; otherwise, a fraction of the medical fluid originally loaded into the nebulizer will remain as ‘residual volume’ within the device by the end of the nebulization period, reducing the net therapeutic benefit [67]. The high values of the aerosol output achieved in the current study indicate that the jet nebulizer employed was capable of aerosolizing the formulations as the energy provided by the nebulizer was high enough to convert the liquid into inhalable aerosol droplets [68]. The high aerosol output attained in the present study agrees with the previous findings reported by Nasr et al., who could achieve about 90% aerosol output upon aerosolization of amphotericin B NE using a Pari LC Sprint jet nebulizer [14]. The second parameter utilized in the current study to evaluate the efficiency of the nebulizer was the aerosol output rate, which demonstrated reasonable values (Table 6). This is because the energy input provided by the nebulizer for atomization was efficient enough to overcome the fluid resistance to the applied shear forces [69]; thus, nebulization of the formulations could be conducted within an acceptable time period. It was reported that the nebulization time is influenced by fluid physicochemical properties like viscosity and surface tension [69]. Results of the current investigation demonstrated that the aerosol output rates of the aerosolized formulations were more or less similar because they possess comparable physicochemical properties, and it was also reported that the performance of jet nebulizers is not highly influenced by the physicochemical properties of the nebulized formulations, in contrast to vibrating mesh nebulizers [29]. The aerosol output rate of the air-jet nebulizer was reported to be superior to that of vibrating mesh nebulizers [29].

**Figure 3:**
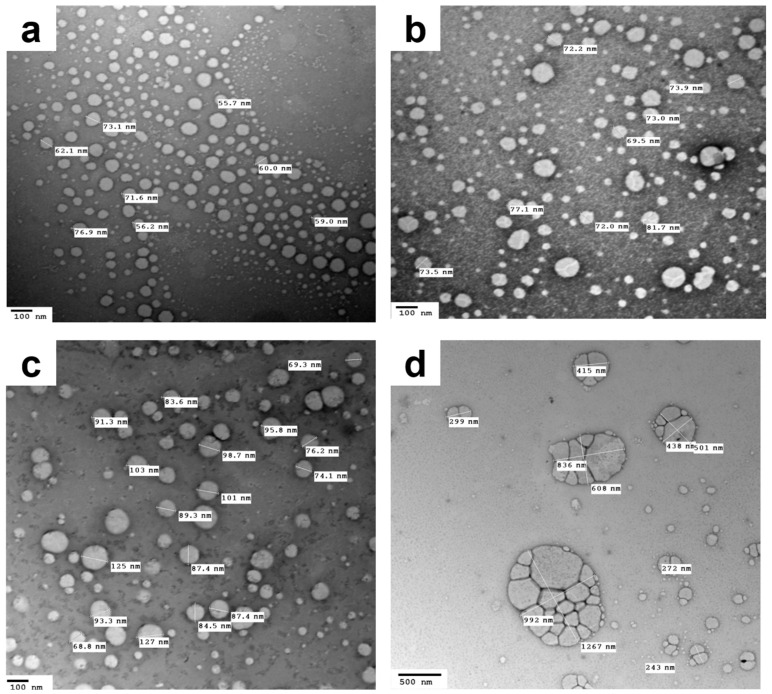
TEM images demonstrating the dispersed phase of the 50-fold saline-diluted NEs of (**a**) C3_TD_, (**b**) C4_TD_ and (**c**,**d**) P2_TD_ after nebulization.

Another important aspect that necessitates studying is the colloidal stability of NEs following nebulization. Thus, for this purpose, the saline-diluted TD-NEs were nebulized, and the colloidal stability was verified in terms of globule size and morphology. Results of DLS measurements revealed a different size distribution pattern for the formulations after nebulization (Table 7), where multiple peaks appeared, including peaks of larger globule size, which were attributed to the aggregation of oil globules during aerosol formation. P2_TD_ exhibited a higher degree of aggregation than the other formulations, as reflected by a higher PdI value (Table 7). However, the majority of the size of the generated globules was in the nanometer size range, unlike the case for the other formulations (Table 7, Figure 3). Many studies showed that the nebulization of colloidal systems would increase their liability to aggregate, and this depends on the nebulizer design [70]. Our results are in accordance with those reported by Laouini et al., in which different carriers were prepared and nebulized for pulmonary delivery of vitamin E. The highest degree of aggregation obtained after nebulization was with NE, while the least was achieved with SLNs. These findings occurred due to the fact that the dispersed phase of SLNs is solid, which is more stable upon aerosolization than the liquid dispersed phase of NEs [70]. In summary, the aforementioned results suggested the potential to aerosolize the fabricated formulations using a jet nebulizer, as manifested by acceptable aerosol output and aerosol output rate, in addition to preserving the globule size of most of the oil droplets within the nanometer range post-nebulization.

### 3.7. Sterilization of NE Formulations

Formulations intended to be administered via inhalation have to be sterile [8]. NEs were subjected to sterilization using two techniques: autoclave and aseptic filtration. Autoclaving is a simple technique compared to aseptic processing that is employed for the sterilization of systems containing thermo-stable drugs. However, the heat applied during autoclaving may affect the formulation’s physical stability [71]. The melting point temperature of TD ranges between 302 and 303 °C [72]; thus, it can maintain stability during autoclaving. Concerning NEs, conflicting results were reported regarding their stability upon autoclaving. Some NEs exhibited phase separation, creaming, color change, and inability to preserve their colloidal stability after steam sterilization, as demonstrated by alteration of the globule size, PdI, and zeta potential [71]. Some authors attributed the unfeasibility of this method for NE sterilization to the failure of non-ionic SAAs to withstand high temperatures [73], while others justified the inconvenience of this method to the potential hydrolysis of lecithins and some lipids, which results in the release of free fatty acids that can adversely affect the NE stability [74]. However, other results revealed that NEs could maintain their colloidal stability after autoclaving, as demonstrated by the inconsiderable change in the globule size, PdI, and zeta potential [75].

NE sterilization via filtration conducted through a 0.22 µm filter is considered a simple and efficient sterilization method, especially for thermolabile drugs [71,76]. However, this sterilization method may influence the globule size distribution and the drug content [71]. It was reported that NE droplets of globule size bigger than 220 nm might block the filter, and loss of the active ingredient may occur due to solute adsorption on the filter [71,76]. However, in the literature, this method appeared to be a more suitable alternative for the sterilization of NEs, which could not resist steam sterilization, as demonstrated by the negligible change in the NE physicochemical properties and reasonable drug recovery following aseptic filtration [71,74].

In the current investigation, the stability of TD-NEs after both sterilization techniques was assessed in terms of globule size and percentage of drug remaining. The results of the globule size analysis are presented in Table 8. Upon comparing the results with the corresponding values before sterilization, it is clearly obvious that there was no substantial change in the mean globule size and PdI after both sterilization techniques, which meant that the formulations could maintain their colloidal stability. On the other hand, upon measuring the percentage of drug remaining, surprisingly, both techniques exhibited similar results, that is, 100% in the case of the Cremophor-based formulations and 93% in case of P2_TD_. These findings indicated that both sterilization techniques did not remarkably affect the drug content of the formulations due to the good incorporation of TD in the formulations. Gue et al. reported at least 90% drug recovery for various active pharmaceutical ingredients after filtration of different drug-loaded NEs through a 0.2 µm filter, which indicated the drug solubility within the developed NEs [77].

The aforementioned data suggested that the two methods seemed convenient for sterilizing the developed formulations, as demonstrated by favorable colloidal stability and acceptable drug recovery after both sterilization techniques.

### 3.8. In-Vitro Cytotoxicity Assessment

**Figure 4:**
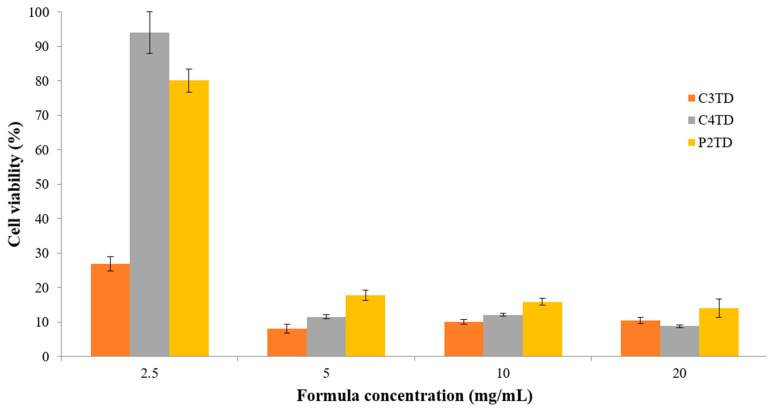
Cytotoxicity testing of various concentrations of TD-NEs against A549 lung cancer cells using MTT assay after 3-h incubation at 37 °C. The results are presented as % cell viability ± S.D.

An optimal drug delivery system has to be biocompatible, biodegradable, and without any adverse effects. In order to determine the safety of TD-loaded NE formulations, a human alveolar adenocarcinoma cell line (A549) was employed, which has been utilized for a long time to evaluate the safety of novel formulations [78]. TD-loaded NEs were serially diluted with DMEM to achieve final test concentrations of 2.5, 5, 10, and 20 mg/mL. MTT assay was used to determine the cellular viability. Results after 3-h incubation are presented in Figure 4. It was observed that 80.1 ± 3.4% cell viability was maintained after incubation of cells with the Poloxamer-based NE formulation at a concentration of 2.5 mg/mL. However, higher concentrations resulted in a sharp reduction in cell viability and were therefore not considered biocompatible. On the other hand, Cremophor EL-based formulation of similar composition C4_TD_ exhibited a comparable cell tolerance limit as demonstrated by a percentage cell viability of 94 ± 6% after incubation with 2.5 mg/mL NE formulation. In contrast, low levels of cell viability were observed throughout the tested concentration range for C3_TD_. The only difference in composition between the formulations C3_TD_ and C4_TD_ was the amount of oil which was 5% higher in the formulation C3_TD_ at the expense of water. The higher amount of oil in the formulation C3_TD_ might have resulted in lower cell viability.

Cremophor EL is a non-ionic SAA with an HLB value of 12–14, capable of solubilizing many hydrophobic sparingly soluble drugs [79]. Cremophor EL is approved for clinical use and is a component of many marketed products, including Taxol^®^ and Sandimmune^®^ for I.V. infusion. Clinical application of Cremophor EL-based formulations has raised concerns related to many SAA-related adverse effects reported, mainly anaphylactoid hypersensitivity reactions [80,81,82]. However, the observed effects are associated with relatively high blood concentrations of Cremophore EL achieved by I.V. infusion of the therapeutic doses of respective drug products; besides, prolonged exposure time of up to 24 h is sometimes dictated by therapeutic protocols. In this study, care was taken during the formulation development phase to employ the least possible SAA concentration needed to achieve the desired pharmaceutical attributes in terms of drug loading capacity and aqueous dilution stability of the formulation. To the best of our knowledge, no previous reports of Cremophor EL-containing formulations for pulmonary use in pediatric PAH exist. As for in-vitro toxicity in cell monolayers, cytotoxic effects are known to be cell and formulation type dependent. The cell tolerable concentration limit of Cremophor EL was reported to be 5 mg/mL in the Caco-2 epithelial cell line down to 0.1 mg/mL in the brain endothelial cell line [81]. In A549 cells, cell tolerable concentration of Cremophor EL was calculated as 50 µg/mL, but importantly, the study tested for the toxicity of Cremophor EL in combination with ethanol at a 1:1 ratio, that is, the vehicle employed in IV Taxol^®^ injections [80]. In this work, the cell tolerable limit detected for C4_TD_ was 2.5 mg/mL, equivalent to 500 µg/mL Cremophor EL, which empathizes the possible modulation of the safety profile of individual excipients depending on the formulation. This is further demonstrated by the rather unexpected finding of low cell viability levels in response to incubation with a similar concentration of C3_TD_ formulation, which differs from C4_TD_ only in the oil content. Further investigation of the detailed mechanism of cell toxicity is needed to elucidate this finding.

On the other hand, many studies showed that Poloxamers exhibited a considerably low toxicity in cultured cells or even cytoprotection, yet, some compounds displayed cytotoxicity [83]. Among the different mechanisms involved in their cytotoxicity is the alteration in the membrane fluidity. It is believed that the higher the HLB value, the higher the hydrophilicity of the compound and the higher its possible insertion into the cell membrane [83]. Although the HLB value of Poloxamer-407 ranges between 18 to 23, it was shown to exert slight changes in the membranes when compared with another type of Poloxamer with a lower HLB value [83]. Poloxamer-407 is included in many commercially available pharmaceutical products as it is biocompatible and is approved by the FDA for clinical use [83]. However, some reports showed that it alters lipid metabolism, but this complication occurs after using high doses [83]. Moreover, there are some conflicting reports regarding its effect on renal elimination [83].

In-vitro biocompatibility of Poloxamer-407 on the A549 cell line was tested [84], and results showed that Poloxamer exhibited negligible toxicity in the concentration ranges (0.1–100 μg/mL). When the concentration of Poloxamer reached 1000 μg/mL, approximately 90% cell viability was attained, in contrast to Tween-80, which exhibited a more significant cytotoxic effect [84]. This demonstrated the in-vitro biocompatibility of Poloxamer-407 with A549 cells. A cytotoxicity study conducted by Dolatabadi et al. using A549 cells to estimate the toxicity of alendronate sodium-loaded SLNs incorporating Poloxamer-407 as an SAA revealed little impact for the formulations on cell viability; it was even lower than that of the pure, intact drug [85].

In the present work, results obtained from the in-vitro cytotoxicity study for both formulations C4_TD_ and P2_TD_ were considered acceptable, considering the intended dilution for nebulizer use besides further dilution dictated by delivery efficiency and bio-facts of the pulmonary route of drug delivery. Given that, a formulation of the final drug concentration of 2000 µg/mL is prepared, diluting at least 300 folds before administration. Then 10% to 30% of this dose will be delivered to the lungs, where it will be diluted by the extravascular lung fluid, which ranges between 3 mL to 10 mL, as discussed before. Thus, it is estimated that the formulation will be exposed to at least 1800 folds of dilution. Based on these calculations, the results of cytotoxicity study suggested the potential safety of both formulations in-vivo. A similar approach was adopted by Shan et al., who considered the dilution of the formulations within the lungs [86].

### 3.9. In-Vivo Lung Toxicity Evaluation

In addition to the in-vitro cytotoxicity study, animal tests were carried out to further evaluate the safety of C4_TD_ and P2_TD_ at the two calculated dosing levels after appropriate dilution with saline. The animals were administered the diluted formulations via the oro-tracheal route, which is a non-invasive pathway to achieve pulmonary delivery without the need for surgical intervention; however, the animal had to be anesthetized. Afterward, a biocompatibility assessment was conducted via biochemical analysis of the BAL fluid and histopathological examination of rat lung tissues.

#### 3.9.1. Biochemical Assessment of BAL Fluid

**Figure 5:**
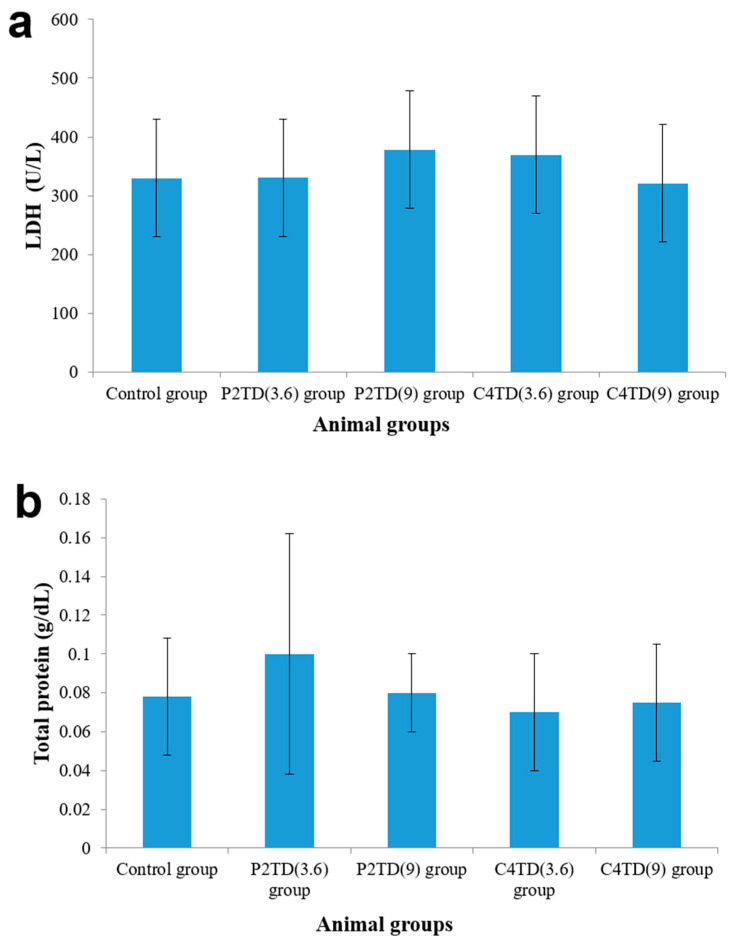
(**a**) LDH and (**b**) total protein content determined in BAL fluid of different rat groups (*n* = 5) after oro-tracheal administration of TD-NEs. The results are presented as ±S.D. All groups were statistically compared to the control group revealing non-significant difference (*p* > 0.05).

In order to determine whether the pulmonary administration of TD-NEs leads to lung damage, BAL studies were carried out. These studies provide a rapid method to examine the damage of the airway epithelial cells through biochemical analysis of the BAL fluid. The presence of high protein content in the fluid indicates variation in the permeability of the alveolar-capillary barrier, in addition to membrane lysis. LDH indicates cell damage or inflammation. The results of the analysis of the biochemical markers are illustrated in Figure 5. Statistical analysis revealed no statistically significant difference in toxicity between the formulation-treated groups and the control group (*p* > 0.05). Therefore, oro-tracheal administration of the two formulations in the two tested doses exhibited comparable biocompatibility to saline in terms of LDH activity and total protein content.

#### 3.9.2. Histopathological Examination

**Figure 6:**
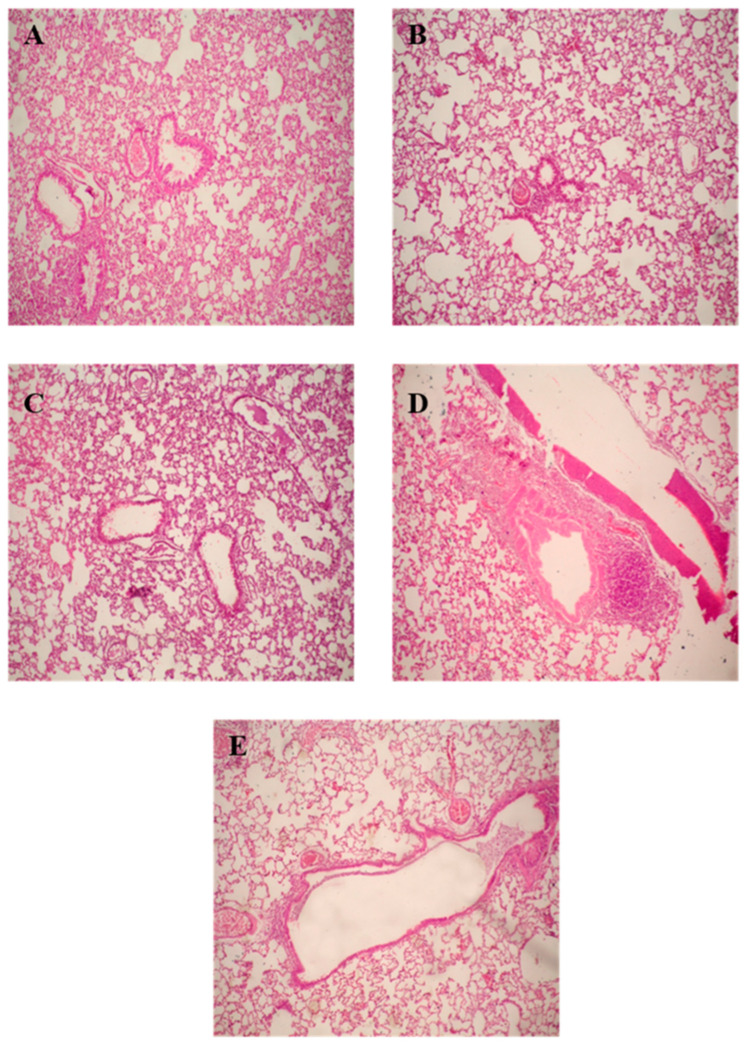
Histopathological photomicrographs of stained lung tissue sections of rats after oro-tracheal administration of different treatments: (**A**) control group, (**B**) P2_TD(3.6)_ group, (**C**) P2_TD(9)_ group, (**D**) C4_TD(3.6)_ group and (**E**) C4_TD(9)_ group.

To detect any pathological change that may occur to the lungs due to the administration of the formulations, a histopathological examination of the stained lung tissues was done. Autopsy lung samples were collected 24-h post-dosing from the test groups and were compared with the control group. Results of histopathological examination are given detailed scores in Table 9 and are also presented in Figure 6. The stained rat lung tissues of the negative control group showed only a few pathological changes after 24 h of oro-tracheal administration of saline. There was no change in the basic lung structure, the microscopic appearance of lung parenchyma was within the normal range, and there was no cellular infiltration or fibrosis. Alveoli could maintain their normal morphology and regular organization, the alveolar wall remained intact, and there was no change in the alveolar surface area. Mild lung edema and mild congestion were only observed in the lungs of rats in this group, which are regarded as a primary immune response to the procedure.

The P2_TD(3.6)_ group revealed subtle and minimal pathological changes analogous to the negative control group (Table 9, Figure 6B). The classical structure of normal lungs was still preserved; however, mild lung edema and mild congestion were observed, comparable to that of the control group. The P2_TD(9)_ group manifested similar results to those of the P2_TD(3.6)_ group, but mild alveolar inflammation, mild cellular infiltration, and mild inflammatory infiltrate in the bronchus were detected in some specimens (Table 9, Figure 6C). This slight increase in toxicity observed in the P2_TD(9)_ group compared with the P2_TD(3.6)_ group was due to the difference in folds of dilution of the formulation, as the formulation administered to the animals of the P2_TD(9)_ group was less diluted than the one administered to the P2_TD(3.6)_ group.

In the case of rats that administered the formulation C4_TD_, results of the histopathological assessment revealed that the formulation toxicity profile in the lower dose (C4_TD(3.6)_ group) was somewhat similar to that of the P2_TD(9)_ group, but cellular infiltration and moderate alveolar inflammation were detected in all the test samples (Table 9, Figure 6D). For animals that were administered the higher dose (C4_TD(9)_ group), exaggeration of the pathological changes was observed, where higher edema, congestion, cellular infiltration, and alveolar inflammation were detected (Table 9, Figure 6E). The degree of inflammation and the severe disturbance of lung architecture observed in the case of animals that were administered C4_TD_ was unacceptable as the formulation is supposed to be administered on daily bases, which may induce further lung inflammation; thus, its toxicity profile does not allow it to be used in clinical practice.

The Poloxamer-based formulation exhibited a better safety profile than the Cremophor-based NE due to the higher biocompatibility of Poloxamer-407. It was reported that pulmonary delivery of 13% *w*/*v* Poloxamer-407 solutions to the deep lungs did not induce any inflammation in the lungs; however, it changed the permeability of the alveolo-capillary barrier, but this alteration was reversible over time [87]. The more diluted Poloxamer-based NE demonstrated higher biocompatibility than the more concentrated formulation, as it was reported that pulmonary delivery of low doses of various Poloxamers to the airways induces no signs of acute or chronic lung toxicity; however, high doses of Poloxamer-407 are pro-inflammatory and should be avoided in pulmonary application [88]. On the other hand, Cremophor EL was reported to induce pulmonary toxicity, as demonstrated by increased levels of LDH, total proteins, and neutrophils in the BAL fluid following intratracheal delivery [89]. Cremophor EL was also reported to trigger hypersensitivity pneumonitis, marked by bronchospasm, urticaria, and angioedema; in addition, it increases the risk of impairing lung surfactant function [90,91]. Thus, C4_TD_ exhibited an unacceptable safety profile when compared with P2_TD_.

In conclusion, data from the in-vivo toxicity study suggested that the formulation P2_TD_ administered at a low concentration did not induce major lung damage. The minor changes detected may be explained as a primary immune response to the formulation in the lung. All the observed changes were very subtle and within the physiological limits. Overall, this data represented the safety profile of the formulation after a single administration, but the long-term safety profile after multiple administrations should be evaluated.

### 3.10. Long-Term Stability Study

Results of the long-term stability study indicated that P2_TD_ was stable under different storage conditions (Table 10), where no change in clarity or drug precipitation was detected upon a visual examination which was further confirmed by %T and percentage drug content values equal to or approaching 100%. Globule size analysis after 50-fold dilution with water indicated that the formulation exhibited no aggregation as the dispersed phase remained in the nanometer range within a narrow size distribution, as reflected by narrow PdI values. In short, the results of the long-term stability study confirm the stability of P2_TD_ for three months upon storage in different conditions, as indicated by the negligible changes in the physicochemical properties.

## 4. Conclusions

In the current study, we managed to fabricate TD o/w NE for the treatment of pediatric PAH via nebulization. Innovatively, the phase diagram and the TD-NE formulation developed by our research group were exploited and optimized via screening of various excipients to prepare NEs that can resist aqueous dilution without exhibiting phase separation or drug precipitation. Accordingly, two-phase diagrams were developed, from which various formulations of different compositions were selected for TD loading. The NEs that can withstand aqueous dilution without destruction were selected, their physicochemical properties were studied, and their suitability for pulmonary application was evaluated. The formulations C3_TD_, C4_TD_, and P2_TD_ demonstrated reasonable TD loading, their droplet size was within the nanometer range, their pH and viscosity after dilution with saline were acceptable for pulmonary application, and they exhibited favorable stability following different stress conditions. The saline-diluted NEs could be efficiently nebulized via a Microlux^®^ jet nebulizer with an aerosol output higher than 90% and an aerosol output rate of 0.2 g/min. Moreover, the NEs could maintain their stability after nebulization and sterilization, both prerequisites for pulmonary drug delivery. The in-vitro cytotoxicity study demonstrated that A549 cells exhibited very good tolerance to C4_TD_ and P2_TD_ formulations. The biocompatibility of the NEs was further evaluated in-vivo following oro-tracheal administration in rats, where the biochemical analysis of BAL fluid and histopathological examination of lung tissues confirmed the safety of P2_TD_ for pulmonary application in low concentrations. However, long-term biocompatibility studies are further required to ensure the potential safety of P2_TD_ after multiple administrations. In addition, evaluation of the biological efficacy of this formulation in therapeutic animal models of PAH is crucial to guarantee the development of a successful formulation. Overall, the presented findings demonstrated that utilizing the Poloxamer-based P2_TD_ NE in low concentrations could be a promising approach for the treatment of pediatric PAH.

## Figures and Tables

**Table 1:** Screening of oily phases with different surfactants (SAAs) and surfactant mixtures (S_mixs_) based on visual inspection.

Surfactant/S_mix_	Capmul + Labrafac (1:1) (*w*/*w*)	Capmul-MCM-EP	Labrafac-Lipophile WL 1349
Labrasol	Turbid	Turbid	Turbid
Cremophor EL	Clear	Clear	Clear
Cremophor RH40	Clear	Clear	Clear
Tween-80	Clear	Turbid	Clear
Poloxamer-407	Turbid	Clear	Turbid
Labrasol:Cremophor EL (1:1)	Clear	Turbid	Turbid
Labrasol:Cremophor RH40 (1:1)	Clear	Turbid	Slightly turbid
Labrasol:Tween-80 (1:1)	Turbid	-	Turbid
Labrasol:Poloxamer 407 (1:1)	Clear	Slightly turbid	Turbid

**Table 2:** Composition of the selected NEs and the corresponding formulation assessment concerning physical appearance after dilution, TD solubility, and drug remaining after dilution.

Formulation Code	System	NE Composition			Physical Appearance after Dilution	Solubility of TD (mg/mL)	TD Remaining after Dilution (%)
		Oil Mixture	Water	S_mix_			
C1	B	10%	30%	60%	Clear	-	-
C2	B	10%	40%	50%	Clear	-	-
C3	B	10%	50%	40%	Clear	3.39	97
C4	B	5%	55%	40%	Clear	3.52	100
C5	B	10%	60%	30%	Turbid	-	-
P1	C	10%	50%	40%	Slightly turbid	-	-
P2	C	5%	55%	40%	Clear	2.45	99

**Table 3:** Physicochemical properties of placebo and TD-loaded NE formulations.

Formulation	Refractive Index	% T	pH	Viscosity (cP)	Zeta Potential (mV)
C3	1.343 ± 0.00	100 ± 0.13	6.169 ± 0.01	7.4 ± 0.40	0.75 ± 0.2
C3_TD_	1.348 ± 0.00	99.6 ± 0.8	5.958 ± 0.01	4.31 ± 0.3	−0.471 ± 0.1
C4	1.343 ± 0.00	100 ± 0.60	5.993 ± 0.03	4.5 ± 0.10	−0.132 ± 0.2
C4_TD_	1.343 ± 0.00	100 ± 0.13	5.890 ± 0.01	7.5 ± 0.30	1.15 ± 0.3
P2	1.347 ± 0.00	100 ± 0.26	5.446 ± 0.02	4.69 ± 0.2	−0.528 ± 0.1
P2_TD_	1.345 ± 0.00	99.6 ± 0.5	5.565 ± 0.03	3.97 ± 0.3	−1.42 ± 0.2

**Table 4:** Globule size distribution by the intensity of placebo and TD-loaded NE formulations after 50-fold dilution with water or saline.

Formulation	Diameter (nm)	% Intensity	PdI
C3 *	21.21 ± 0.32	98.5 ± 1.7	0.157 ± 0.020
C3_TD_	19.30 ± 0.04	100	0.075 ± 0.002
C3_TD(S)_	18.77 ± 0.08	100	0.031 ± 0.004
C4	16.51 ± 0.14	100	0.097 ± 0.010
C4_TD_	16.03 ± 0.28	100	0.068 ± 0.020
C4_TD(S)_	15.96 ± 0.10	100	0.048 ± 0.004
P2	25.47 ± 0.01	100	0.042 ± 0.006
P2_TD_	25.04 ± 0.20	100	0.027 ± 0.016
P2_TD(S)_	26.38 ± 0.35	100	0.065 ± 0.018

(*): formulations showed multimodal size distribution. C3 *: a second peak of size 2918 nm ± 2529 (1.5% ± 1.7) was detected. (s): formulations diluted with saline.

**Table 5:** Globule size distribution by intensity of 50-fold diluted TD-loaded NE formulations after accelerated stability tests.

Accelerated Stability Test	Formulation	Diameter (nm)	% Intensity	PdI
Initial size distribution	C3_TD_	18.66 ± 0.05	100	0.042 ± 0.010
	C4_TD_	15.43 ± 0.05	100	0.033 ± 0.008
	P2_TD_	25.74 ± 0.05	100	0.024 ± 0.005
Centrifugation	C3_TD_	19.03 ± 0.16	100	0.050 ± 0.010
	C4_TD_	15.5 ± 0.09	100	0.037 ± 0.090
	P2_TD_ *	28.91 ± 0.8	96.8 ± 0.7	0.197 ± 0.019
Freeze-thaw cycles	C3_TD_ *	33.19 ± 1.18	99.2 ± 1	0.204 ± 0.006
	C4_TD_	15.35 ± 0.07	100	0.031 ± 0.010
	P2_TD_	44.56 ± 0.9	100	0.181 ± 0.003

(*) formulations showed bimodal size distribution. P2_TD_ *: a second peak of size 4793 nm ± 164.8 (3.2% ± 0.7) was detected. C3_TD_ *: a second peak of size 1523 nm ± 2633 (0.8% ± 1) was detected.

**Table 6:** Nebulization performance of 50-fold saline diluted TD-NEs using Microlux^®^ jet nebulizer.

Formulation	Aerosol Output (%)	Aerosol Output Rate (g/min)
C3_TD_	94.4 ± 1.84	0.19 ± 0.003
C4_TD_	96.8 ± 1.19	0.20 ± 0.002
P2_TD_	96.5 ± 0.68	0.20 ± 0.002

**Table 7:** Globule size distribution by intensity of 50-fold saline diluted TD-loaded NE formulations before and after nebulization.

Condition	Formulation	Peak 1		Peak 2		Peak 3		PdI
		Diameter (nm)	% Intensity	Diameter (nm)	% Intensity	Diameter (nm)	% Intensity	
Before nebulization	C3_TD_	18.77 ± 0.08	100	0	0	0	0	0.031 ± 0.004
	C4_TD_	15.96 ± 0.11	100	0	0	0	0	0.048 ± 0.004
	P2_TD_	26.38 ± 0.35	100	0	0	0	0	0.065 ± 0.018
After nebulization	C3_TD_	19.22 ± 0.23	96.43 ± 2.5	3530.6 ± 1668	3.56 ± 2.50	0	0	0.203 ± 0.04
	C4_TD_	15.01 ± 0.59	91.90 ± 1.2	1915.7 ± 2235	7.03 ± 0.35	1673.3 ± 1670	1.1 ± 0.98	0.24 ± 0.015
	P2_TD_	28.53 ± 1.15	44.43 ± 3.8	192.70 ± 5.40	54.4 ± 3.60	2730 ± 936.50	1.13 ± 0.3	0.49 ± 0.020

**Table 8:** Globule size distribution by intensity of 50-fold diluted TD-NEs after sterilization.

Formulation	Autoclave			Filtration		
	Diameter (nm)	% Intensity	PdI	Diameter (nm)	% Intensity	PdI
C3_TD_	18.81 ± 0.19	100	0.040 ± 0.017	19.98 ± 0.11	100	0.063 ± 0.007
C4_TD_	15.36 ± 0.04	100	0.026 ± 0.014	15.21 ± 0.03	100	0.044 ± 0.005
P2_TD_	25.49 ± 0.20	100	0.023 ± 0.014	25.43 ± 0.07	100	0.034 ± 0.010

**Table 9:** Detailed histopathological assessment of rat lung tissues after oro-tracheal administration of different treatments.

Points of Assessment	Control Group	P2_TD(3.6)_ Group	P2_TD(9)_ Group	C4_TD(3.6)_ Group	C4_TD(9)_ Group
Basic lung structure	Normal	Normal	Normal	Normal	Normal
Microscopic appearance of lung parenchyma	Normal	Normal	Normal	Normal	Normal
Lung edema	+	+	+	+	++
Inflammatory infiltrate in bronchus	-	-	+	+	+
Congestion	-	+	+	+	++
Lung inflammation:					
Cellular infiltration	-	-	+/-	+	+/++
Fibrosis and granuloma in the perivascular region	-	-	-	-	-
Alveoli					
Morphology	Normal	Normal	Normal	Normal	Normal
Alveolar wall	Intact	Intact	Intact	Intact	Intact
Intralveolar septa	Normal	Normal	Normal	Normal	Normal
Alveolar surface area	Normal	Normal	Normal	Normal	Normal
Organization of alveoli	Regular	Regular	Regular	Regular	Regular
Alveolar inflammation	-	-	+/-	+	++

(-) denotes no pathological change, (+/-) pathological changes were detected in some specimens, (+) pathological changes were detected in all specimens, (++) intense pathological changes were detected in all specimens.

**Table 10:** Results of long-term stability study of P2_TD_ NE formulation at different storage conditions.

Period	Storage Condition	Visual Examination	Globule Size			Drug Content (%)	% T
			Diameter (nm)	% Intensity	PdI		
Initial	-	Clear	25.6 ± 0.5	100	0.046 ± 0.01	100	98.6 ± 0.8
First month	RT	Clear	26.84 ± 0.4	100	0.1 ± 0.03	97.8	99.5 ± 0.001
	Refrigerator	Clear	27.61 ± 0.27	100	0.113 ± 0.02	99.2	98.6 ± 0.001
	40 °C/75% RH	Clear	25.4 ± 0.07	100	0.084 ± 0.04	100	98.8 ± 0.002
Second month	RT	Clear	25.28 ± 0.09	100	0.034 ± 0.009	97.8	100 ± 0.001
	Refrigerator	Clear	24.91 ± 0.1	100	0.024 ± 0.01	100	98.4 ± 0.002
	40 °C/75% RH	Clear	24.98 ± 0.1	100	0.01 ± 0.009	100	99 ± 0.001
Third month	RT	Clear	25.43 ± 0.07	100	0.042 ± 0.007	100	100
	Refrigerator	Clear	25.33 ± 0.1	100	0.032 ± 0.006	100	100
	40 °C/75% RH	Clear	26.29 ± 0.3	100	0.063 ± 0.01	100	100

## Data Availability

Not applicable.
